# Effects of Extrusion Treatment on the Physicochemical and Baking Quality of *Japonica* Rice Batters and Rice Breads

**DOI:** 10.3390/gels11020086

**Published:** 2025-01-22

**Authors:** Wenxia He, Jingni Tang, Yang Chen, Guanhui Liu, Zhenni Li, Jie Tu, Yixuan Li

**Affiliations:** 1 School of Grain Science and Technology, Jiangsu University of Science and Technology, Mengxi Road 2, Zhenjiang 212003, China; hewenxia1012@163.com (W.H.); 212241821113@stu.just.edu.cn (J.T.); 231211803112@stu.just.edu.cn (Y.C.); jeany@just.edu.cn (Z.L.); yixuanli@just.edu.cn (Y.L.); 2 School of Biotechnology, Jiangsu University of Science and Technology, Changhui Road 666, Zhenjiang 212100, China; tujie@just.edu.cn; 3 Jiangsu Provincial Research Center for Grain Bioprocessing Engineering, Zhenjiang 211102, China

**Keywords:** *japonica* rice flour, extrusion treatment, batter, rice bread, amylose, gelatinization degree

## Abstract

Gluten-free rice bread made from *japonica* rice finds challenge in achieving a good shape and structure, presenting a significant obstacle in the baking industry. This study aims to improve the quality of rice bread with *japonica* rice flour by hot extrusion treatment (without additives). The effects of extrusion on the amylose content, gelatinization degree, hydration capacity, short-range molecular ordering, and microstructure of *japonica* rice flour were investigated. The results show that the amylose content of the extruded flour increased by 12.43% and the gelatinization degree of it increased by 13.23 times, showing disrupted starch granules, numerous pores, and a better hydration capacity. The addition of extruded flour improved the overall viscoelasticity of the batter. Compared to the control group, the specific volume and porosity of the optimized rice bread were increased by 19.46% and 61.92%, respectively. The gas cell density was increased by 4.63 times, and the average gas cell area of rice bread was reduced by 47.14%. The correlations among the raw material properties of rice flour, the batter properties, and the quality of rice bread products were revealed by principal component analysis. This study demonstrates that the addition of moderate amounts of extruded *japonica* rice flour could improve the quality of rice bread products.

## 1. Introduction

Coeliac disease is a chronic, multi-organ autoimmune disease that affects the small bowel in genetically predisposed persons, precipitated by the ingestion of gluten. Gluten is a type of protein mainly found in wheat, which is mainly composed of glutenin and alcohol-soluble gliadin [1]. The only effective and safe treatment for people with celiac disease is adherence to a strict gluten-free diet [2]. Gluten intolerance is a condition characterized by irritable bowel syndrome (IBS)-like symptoms and extra-intestinal manifestations, occurring in a few hours or days after the ingestion of gluten-containing food, improving rapidly with gluten withdrawal and relapsing soon after gluten challenge. In recent years, the per capita consumption of wheat has decreased by 9% globally, while the number of consumers avoiding gluten consumption has been increasing year by year [3]. At the same time, the pursuit of baked products is not limited to traditional ingredients and flavors, but also desired breakthroughs in nutrition, taste, and innovation. As a result, the gluten-free bakery market is expanding rapidly, with a global market size exceeding USD 5 billion. Rice is one of the important grains consumed globally. Due to the high biological value and hypoallergenic property of rice protein, rice has become a raw material for baby food and health food. Among various gluten-free ingredients, rice is considered one of the most suitable for bakery products due to its hypoallergenic, easy-to-digest, and low-fat properties [4]. However, the absence of gluten in rice presents a challenge in forming a viscoelastic network within the batter, hindering the retention of gases produced during fermentation. This significantly impacts the specific volume and textural properties of rice-based bread [5].

*Oryza sativa* subsp. *Japonica* is an important subspecies of *Oryza sativa*. In China, *japonica* rice accounts for more than 30% of total rice production. This semi-glutinous variety is characterized by a low amylose content (typically around 10%) and low protein content (around 8%), which contributes to low hardness, high viscosity, and better edible quality [6]. However, high levels of amylose have been recognized as a key factor influencing the baking quality of bread [7]. For instance, Aoki et al. [8] demonstrated that rice with up to 22.3% amylose content could be used to prepare 100% rice bread with enhanced gas-retention properties and a high specific volume. The dual factors of low amylose content and low protein content constrain the production of baked products from *japonica* rice (including broken rice produced during milling). Therefore, it is necessary to find a processing method that can produce high-quality *japonica* rice bread.

Several studies have shown that the structural properties of rice bread can also be improved by incorporating hydrocolloids, enzymes, and emulsifiers, resulting in a texture similar to that of wheat bread [9,10]. However, consumer resistance to additives remains a concern. Compared to chemical treatments, physical treatments offer ad-vantages in terms of speed, efficiency, sustainability, and health benefits. For example, Saito et al. [11] developed a method to produce additive-free rice bread using only hot water and flour. Extrusion processing, a physical technique involving the high-temperature, high-pressure, and high-shear treatment of cereal flours, has been shown to alter starch structures rapidly [12]. In recent years, extrusion has become a widely used method for producing various starchy foods, including noodles (extruded at 70–120 °C) and breakfast cereals (extruded at 60–140 °C) [13,14,15]. During hot extrusion processing, starch undergoes gelatinization and degradation, leading to an increase in amylose content and a decrease in amylopectin levels [16]. Li et al. [17] demonstrated that the extrusion of modified buckwheat flour disrupted the granular structure of starch and facilitated the formation of a starch network. This enhanced the stability and viscoelasticity of the dough, significantly improving the texture of the noodles. These findings suggest that extrusion treatment could provide a theoretical foundation for improving the quality of rice bread. However, the applicability of extrusion to the production of *japonica* rice bread and the identification of suitable quality evaluation criteria still required further investigation. Therefore, this study uses *japonica* rice as a raw material to explore the effects of extrusion treatment on the physicochemical properties of *japonica* rice flour and batter, as well as the quality of rice bread. Additionally, principal component analysis (PCA) will be employed to examine the relationships among key indicators in the molding process of *japonica* rice bread, with the goal of providing both a theoretical framework and experimental basis for the development of natural *japonica* rice bread products.

## 2. Results and Discussion

### 2.1. Effect of Extrusion Treatment on the Physicochemical Properties and the Degree of Gelatinization of Rice Flour

Table 1 showed that the starch content of the rice flour decreased by 4.03% after extrusion treatment. With high temperature, high pressure, and high shear, starch underwent gelatinization and degradation. The degradation of starch mainly occurred on amylopectin, generating small molecules of sugar and dextrin. In addition, the formation of starch-fat complexes occurred during extrusion [18]. The extrusion treatment increased the amylose content of the rice flour by 12.43%. With the action of high shear, the α-1,6 glycosidic bonds of branched starch were broken, resulting in a significant (*p* < 0.05) increase in the content of amylose [19], which was similar to the results of Liu et al. [20], who studied extrusion treatment of rice flour.

The water absorption index (WAI) represents the hydrophilicity of a sample, which mainly reflects the gelatinization and water-holding capacity of starch in food; the water solubility index (WS) represents the solubility of a sample, which mainly reflects the degree of degradation and destruction of macromolecules. The WAI and WS of the rice flour were significantly (*p* < 0.05) improved by extrusion treatment, and its hydration capacity was significantly (*p* < 0.05) increased. The WAI and WS values of the extruded flour were increased by 2.14 and 8.54 times, respectively. During extrusion, this may have been due to the shear force disrupting the ordered structure and granule morphology of starch, as well as the hydrogen bonding between the hydroxyl groups of the double helix of the starch molecule, facilitating the diffusion of the water molecules into the extruded starch granules [7], which all increased the WAI and WS values. An increase in WAI indicates an increase in the gelatinization and water holding capacity of extruded flour, and an increase in WS indicates an increase in the degree of degradation of starch molecules and the degree of breakage [21]. The hydration capacity of rice flour was also correlated with the quality of fermented products. Qin et al. [22] reported that the addition of 4% to 6% broken starch resulted in an increased hydration capacity of rice flour and increased the elasticity, organization structure, and specific volume of rice bread.

Compared with that of the *japonica* rice flour, the degree of gelatinization of the extruded flour increased by 13.23 times (Table 1), which is consistent with the changes in the hydration capacity of the extruded flour. Under the action of the high shear force of extrusion, the hydrogen bonds of starch will be broken, certain pores will form, and the extruded flour will be mixed with water to be fully pasted [23].

### 2.2. Effect of Extrusion Treatment on the Microstructure of Rice Flour

**Figure 1:**
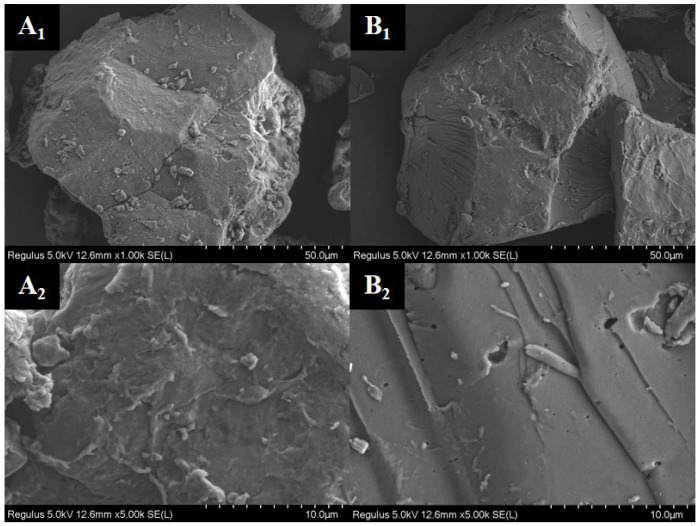
Effects of extrusion treatment on the microstructure of *Japonica* rice flour ((**A_1_**): 1000×, (**A_2_**): 5000×) and extruded flour ((**B_1_**): 1000×, (**B_2_**): 5000×).

The surface morphology and characteristics of starch granules were observed by scanning electron microscopy (QUANTA 250 FEG, Thermo Fisher Scientific, Waltham, MA, USA). Figure 1 shows the microstructures of the *japonica* rice flour and extruded flour. At 1000× magnification (Figure 1(A_1_,B_1_)), the *japonica* rice flour had a rough surface with many starch granules and non-starch components. While, in the extruded rice flour, starch was gelatinized, and the starch granule structure on the surface of the flour was partially destroyed, resulting in a reduction in the amount of small-molecule starch on its surface and an inhomogeneous surface. The above phenomenon is similar to the findings of Zhang et al. [24], who analyzed the surface morphology of extruded brown rice flour.

Further observation at 5000× magnification (Figure 1(A_2_,B_2_)) revealed that the extruded flour had a lamellar reticular structure with distinct pores. This might be due to the absorption of water, swelling, and degradation of the starch granules in the high-temperature environment, leading to destruction of the granule morphology and the formation of many pores. The presence of pores might loosen the internal structure of starch to form a lamellar structure [12]. Wang et al. [14] extruded *indica* rice flour and found that the internal reticulation of starch was enhanced with the presence of many pores and bubbles, leading to an increase in the hydration capacity. The above report is similar to the results of our study.

### 2.3. Effect of Extrusion Treatment on the FTIR of Starch in Rice Flour

**Figure 2:**
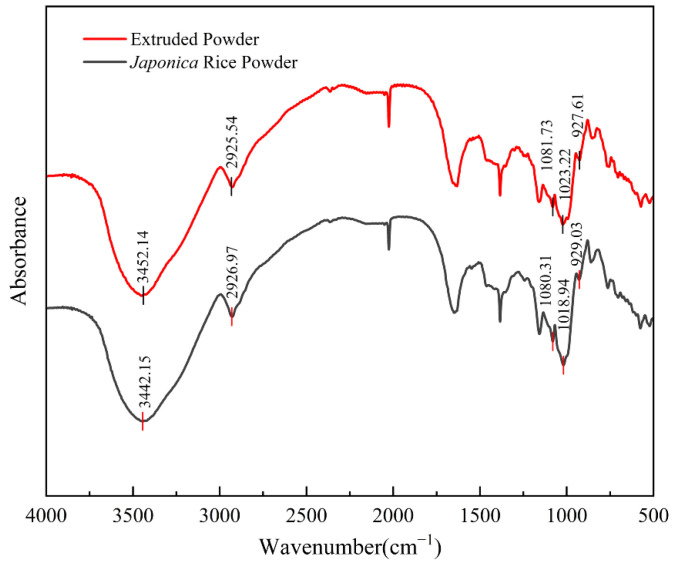
Effects of extrusion treatment on the short-range ordered structure of starch in rice flour.

The short-range ordered structure of starch can be observed by FTIR analysis. The FTIR spectra (500~4000 cm^−1^) of the extruded flour and *japonica* rice flour are shown in Figure 2. The absorbances of *japonica* rice flour at 3442.15 cm^−1^ and 2926.97 cm^−1^ were induced by -OH and C-H vibrations, respectively [25]. Compared with those of *japonica* rice flour, the characteristic absorption peaks of extruded flour did not change significantly (*p* < 0.05). This indicates that there were not any newly produced functional groups in the extruded flour and that the short-range ordered structure of starch in the extruded flour only involved the reorganization of starch molecules or a change in interchain hydrogen bonding. This finding is similar to that of Zhang et al. [24], who studied extrusion treatment of brown rice flour. The absorbances at 1080.31 cm^−1^ and 1081.73 cm^−1^ were related to the crystalline structure of starch, the absorbances at 1018.94 cm^−1^ and 1023.2 cm^−1^ were related to the vibrational mode of the amorphous phase in starch granules, and the absorbances at 929.03 cm^−1^ and 927.61 cm^−1^ described the structure of hydrogen bonds between starch molecules [26]. Generally, a ratio of 1080.31 cm^−1^/1018.94 cm^−1^ or 1081.73 cm^−1^/1023.2 cm^−1^ is used to indicate the degree of short-range ordering of the starch structure, and a ratio of 929.03 cm^−1^/1018.94 cm^−1^ or a ratio of 927.61 cm^−1^/1023.2 cm^−1^ is used for the degree of the double helix in the starch structure [24]. As shown in Figure 2, the short-range ordering values of the starch structures of japonica rice flour and extruded flour were 1.07 and 1.05, respectively, and the double helix degrees of the starch structures were 1.25 and 1.18, respectively. The above results reveal that the degree of order and number of double helices in the extruded flour were reduced, indicating that the extrusion treatment reduced the crystallinity of the starch in the rice flour and disrupted the double-helix structure. This finding is similar to the results of the FTIR analysis of extruded *indica* rice flour reported by Wang et al. [14].

### 2.4. Effect of Extrusion Treatment on Batter-Foaming Properties

**Figure 3:**
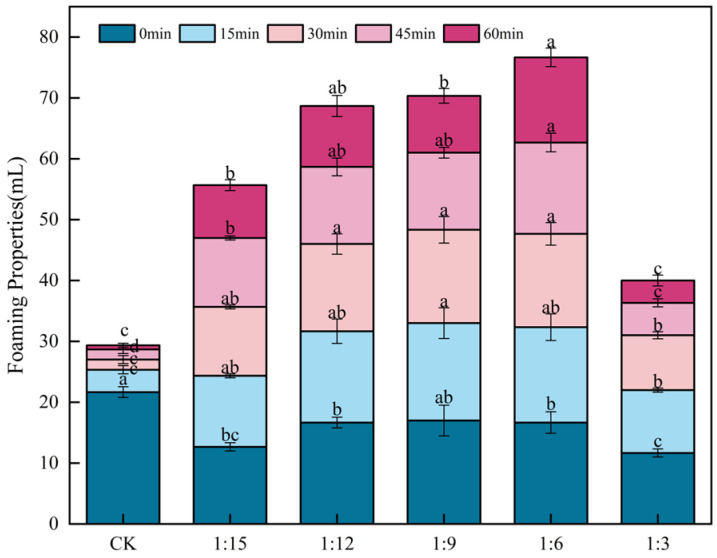
Effects of extrusion treatment on the foaming properties of batter. Different letters (^a–d^) within the same time period indicate significant differences in mean values (*p* < 0.05).

As shown in Figure 3, at 0 min, the foam volume of the CK group (control check) was significantly (*p* < 0.05) greater than that of the other groups. This result was supported by the study of Silvestre [27], whose foam volume of chickpea flour at 0 min was significantly (*p* < 0.05) greater than that of extracts. However, the foam volume of each group gradually decreased with time, and at 15 min, the foam volume of the CK group was significantly (*p* < 0.05) reduced, with a loss of 5.9 times, which indicated that the foaming stability of the CK group was poor. Compared with the CK flour, the addition of extruded flour significantly (*p* < 0.05) improved the foam stability of the batter. Among them, the flour with a ratio of 1:6 had the largest foam volume and the best foaming stability, and only 16.00% of the foam volume was lost after 60 min of mixing. Pycarelle et al. [28] reported that the gas-liquid interfacial stability of flour was positively correlated with the foaming properties of sponge cake batter. This study revealed that the best gas-liquid interfacial stability of the lyophilized batter flour was achieved with a ratio of 1:6, which implies that the foaming properties of the raw material would be improved.

### 2.5. Effect of Extrusion Treatment on the Fermentability of Batters

**Figure 4:**
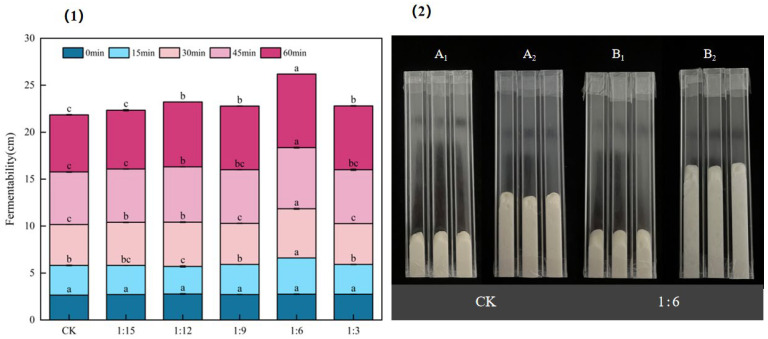
Effects of extrusion treatment on the fermentability of batter. (**1**) Fermentability of batter within 60 min. (**2**) The fermentability of the batter in the CK group (A_1_: 0 min, A_2_: 60 min) and the 1:6 group (B_1_: 0 min, B_2_: 60 min). Different letters ^(a–c)^ within the same time period indicate significant differences in mean values (*p* < 0.05).

The fermentability of a batter affects the organization structure and quality of the final product. Figure 4 shows the effect of extrusion treatment on the fermentability of the batter. After yeast fermentation for gas production, the volume of the batter significantly (*p* < 0.05) increased in all six groups, among which the best fermentation was achieved in the group with a 1:6 ratio of extruded flour to *japonica* rice flour. At 30 min, the height of the batter in the 1:6 ratio group increased by 1.90 times, and at 60 min, the height of its batter increased by 2.85 times. This was because starch was degraded to produce small molecules of sugar after the extrusion treatment, which favored yeast fermentation and led to an increase in yeast gas production [29]. Therefore, the 1:6 ratio resulted in better fermentability, which is consistent with the aforementioned results of better foaming properties.

### 2.6. Effects of Extrusion Treatment on the Rheological Properties of Batters

**Figure 5:**
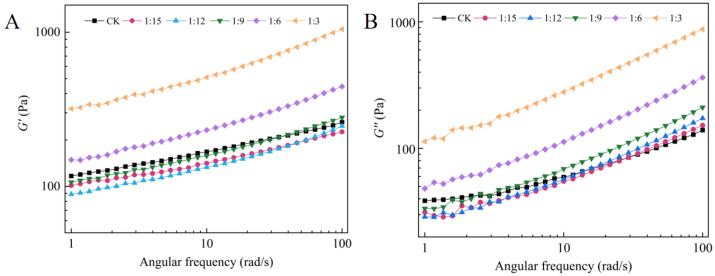
Angular frequency sweep data for different batters. (**A**) Elastic modulus *G′* versus angular frequency. (**B**) Viscous modulus *G″* versus angular frequency.

As shown in Figure 5, both the elastic modulus (*G′*) and the viscous modulus (*G″*) of the batters increased gradually with frequency in the angular frequency range of 0~100 rad/s with a certain degree of dependence, and at the same time, *G′* was greater than *G″* for all the samples, which indicated that elasticity was dominant in the batter system [30]. Both moduli (*G′* and *G″*) are frequency dependent and do not intersect each other, a behavior that can be classified rheologically as a weak gel [31]. Compared with those in the CK group, the *G′* and *G″* values of the batters in each group increased gradually with increasing amounts of extruded flour, indicating that the added extruded flour improved the viscoelasticity of the mixtures. This is because extrusion treatment leads to starch gelatinization, the swelling of starch granules, and an increase in the content of amylose, and the gelatinized starch granules reinforce the amylose to form a gelatinous reticulation [32]. The high content of amylose in the rice flour in the rations group was more likely to result in the formation of a reticulated structure, which affected the *G′* value of the batter. A similar study by Fu et al. [33] used pasted maize starch in place of some wheat flour. Moreover, the extrusion process resulted in the formation of complexes such as starch-protein and starch–fiber complexes, which also contributed to the formation of a more stable structure of the batter, thus increasing the *G′* and *G″* of the batter [17]. In general, batters with high viscoelastic moduli had better air-holding properties during mixing, blending, and fermentation [34]. Highly elastic batters are suitable for the preparation of highly elastic foods such as steamed rice cakes and rice bread [35]. For example, hydroxypropyl methyl cellulose (HPMC) is a hydrophilic colloid commonly used for the preparation of rice bread, and Srikanlaya et al. [29] significantly (*p* < 0.05) increased the *G′* and *G″* of dough by the addition of HPMC to prepare rice bread with uniform porosity comparable to the volume of wheat bread. However, *G′* and *G″* excessively increased when the ratio was 1:3 compared with those of the other groups, and this excessive increase led to a limited fermentability of the batter, which resulted in a decrease in the air-holding capacity of the batter and, ultimately, the preparation of rice bread with a lower specific volume [36].

### 2.7. Effect of Extrusion Treatment on the Baking Quality of Rice Bread

#### 2.7.1. Specific Volume and Tissue Texture of Rice Bread

**Figure 6:**
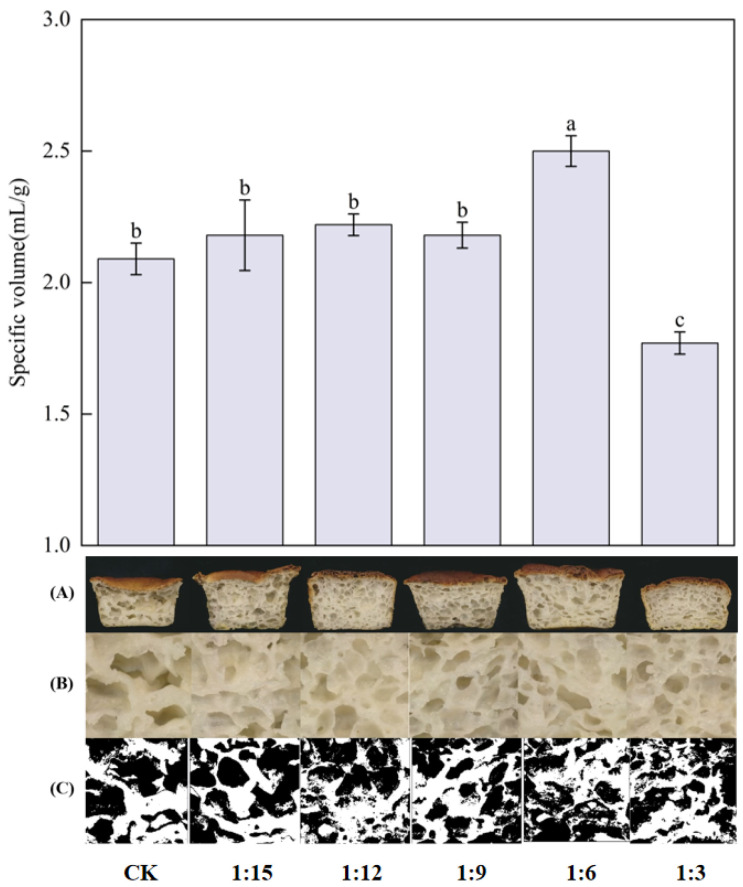
Effects of extrusion treatment on the specific volume and organization structure of the rice bread tissue. (**A**) Bread cross section. (**B**) Bread slice plan view. (**C**) Gray-white analytical diagram of bread slices. Different letters ^(a–c)^ indicate significant differences (*p* < 0.05).

The specific volume of rice bread is shown in Figure 6, and the specific volume of the bread in the CK group was 2.09 mL/g, which was similar to the results of Aoki et al. [8], who prepared rice bread from *japonica* rice flour as a raw material. Compared with the CK group, the highest specific volume of bread was observed when the ratio was 1:6, as it increased by 19.46%. This occurred because extrusion increases the content of amylose, which improves the viscoelasticity of the batter and results in a stable reticulated structure. Similar results were obtained by Martínez et al. [21] when they studied wheat flour bread. As shown in (A), (B), and (C) of Figure 6 and Table 2, the internal pores in the rice bread enriched with extruded flour were more uniform than those in the CK group. Among them, the 1:6 and 1:3 ratio breads had better pore structures. Compared with that in the CK group, the porosity of the bread in the 1:6 ratio group increased by 61.92%, the gas cell density increased by 4.63 times, and the average area of gas cell decreased by 47.14%, which shows that the addition of extruded flour could make the gas cell denser and more uniform and improve the pore structure of the bread. This finding was similar to the results of Rico et al. [37]. The above results reveal that a denser and stronger mesh structure formed in the bread system with the addition of an appropriate amount of extruded flour, thus increasing the specific volume and porosity of the bread. However, the breads in the 1:3 ratio group presented greater porosity and gas cell density and a significantly (*p* < 0.05) lower specific volume of 1.77 mL/g. The specific volume of the bread decreased when the gelatinization of the ingredients was too high [8]. It was shown that pasteurized amylose was degraded by extrusion treatment, and this degradation led to a reduction in the length of the amylose chains [38]. The specific volume of bread made from rice flour decreased when there was an excess of amylose with short molecular chains [39].

#### 2.7.2. Texture of Rice Bread

Table 3 shows the textural properties of the rice breads. It is clear that the rice breads in the CK group present greater hardness values and chewiness properties. With the addition of extruded flour, the hardness and chewiness of the bread reduced, resulting in a softer organization structure. This is attributed to the improved starch gelatinization by extrusion and the effective retention of gas during the fermentation process of the batter [40]. Extrusion also disrupted the ordered structure of the starch, leading to increased water absorption by the rice flour, and the batter became stickier and more gelatinous, which also led to a decrease in the hardness and chewiness of the bread. The result of the texture is consistent with the results of the WS described above. Cohesiveness is a quantification of the internal cohesive structure of rice bread; breads with high viscoelasticity have greater internal cohesion [2], and breads made from the 1:6 ratio flour group had greater cohesion and were more palatable during chewing [41]. There was no significant change in the springiness or resilience of the breads.

### 2.8. Sensory Evaluation of Rice Bread

**Figure 7:**
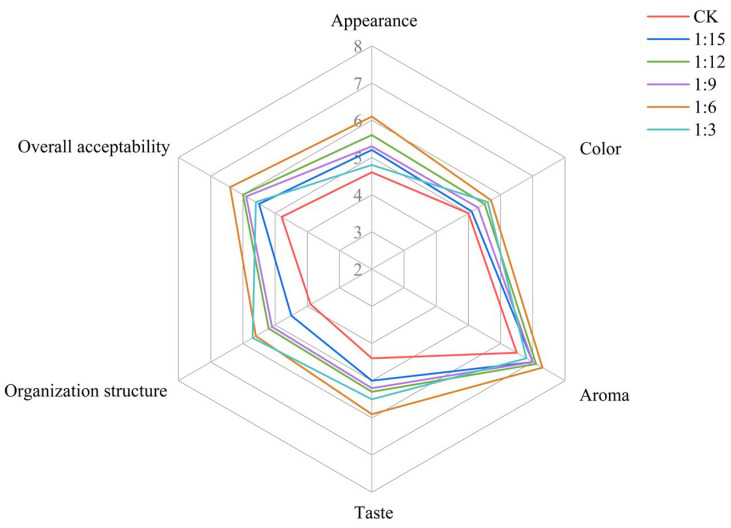
Effect of extrusion treatment on the sensory evaluation of rice breads.

The sensory evaluation of the rice bread is shown in Figure 7. Compared with the CK group, the addition of extruded flour increased the overall acceptability of the rice bread, with the best overall acceptability (6.4 points) and the most popular bread in the 1:6 ratio group. Moreover, the appearance, organization structure, and taste of the bread significantly (*p* < 0.05) improved with the addition of extruded flour, and the color and aroma of the crust slightly improved. Furthermore, the 1:3 ratio group had the best organization of bread, while the appearance of this bread was poorer, indicating that the addition of excess extruded flour affected the appearance of the bread. Therefore, the results indicate that bread enriched at a ratio of 1:6 was the best choice.

### 2.9. Principal Component Analysis (PCA)

**Figure 8:**
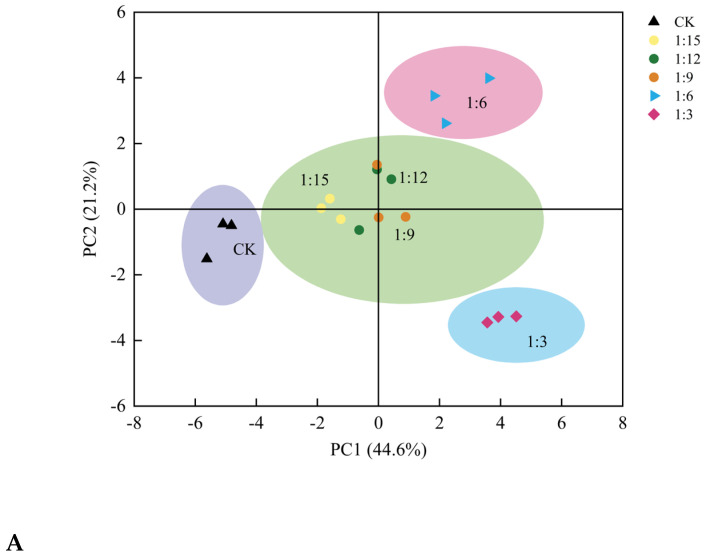

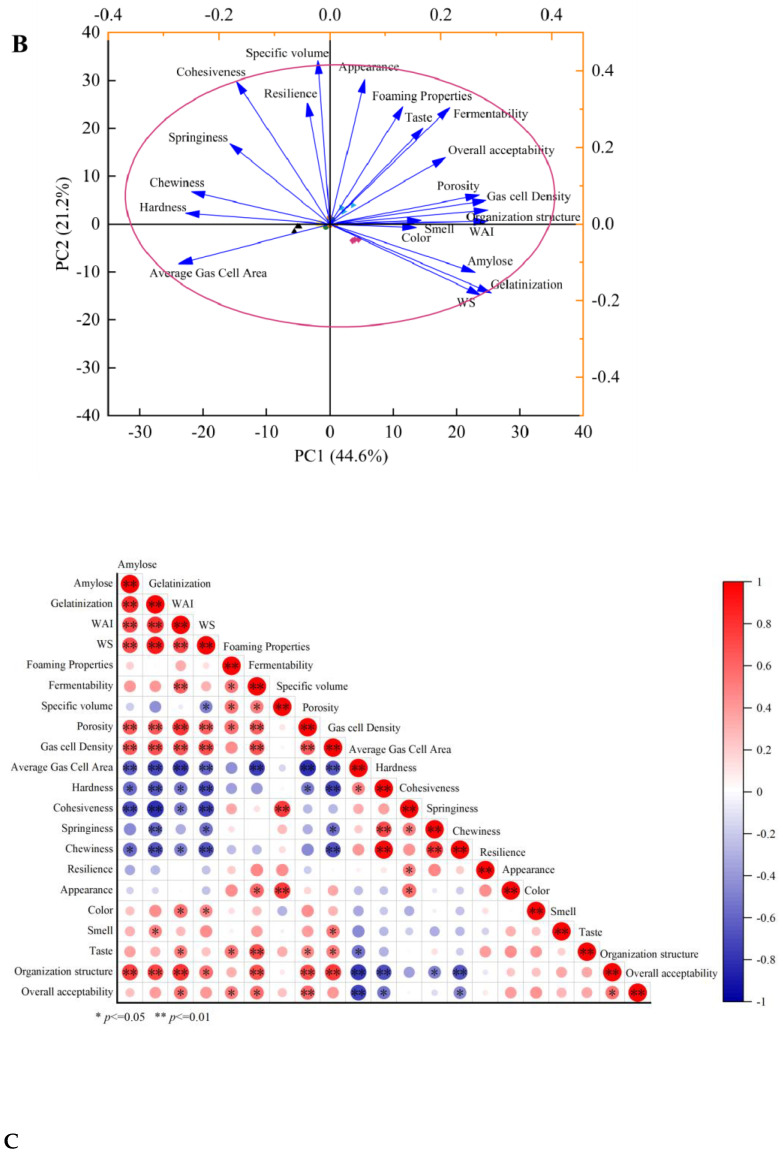
Score plot (**A**), biplot (**B**) and correlation plot (**C**) resulting from principal component analysis on 21 variables determined in rice bread.

The results of the principal component analysis of the indicators in the bread-molding process are shown in Figure 8. The cumulative contribution of the first two principal components constituted 65.8 of the total variance, where the first principal component contained the properties of the raw material (amylose content, gelatinization degree, WAI, WS), fermentation characteristics of the rice batter (foaming stability and fermentability), baking properties of the rice bread (porosity, gas cell density, average gas cell area), and sensory evaluation (taste, organization, and overall acceptability), with a contribution of 44.6% (PC1). The second principal component contained a specific volume, cohesiveness, resilience, and appearance, which could be summarized as bread physical property indicators with a contribution of 21.2% (PC2). The PCA model is usually chosen as the separation model when the contribution rate reaches 60% [42]. The results of the principal component analysis (PCA) indicate that the six groups of rice breads were distinctly separated into four clusters in the two-dimensional space, with significant differences observed. This suggests that the extrusion treatment exerted a specific effect on the quality of both the *japonica* batter and the bread (Figure 8A). Figure 8B presents the relationships between the different groups and the corresponding indicators. The 1:6 group was located in the first quadrant, which indicates that at this ratio, the rice flour had a relatively high amylose content; good foaming stability, WAI and WS, and leavening ability; high specific volume, porosity, and gas cell density; small average area of the gas cell; good hardness, springiness, and chewiness; and that the bread had the highest sensory evaluation and the best overall acceptability, suggesting that breads with a high specific volume and low hardness had a better organizational structure, were uniformly organized, and were the most popular. The CK group was located in the third quadrant, and this group had the weakest batter-foaming stability and fermentability, poor baking quality, and the worst sensory evaluation, suggesting that breads with poor fermentability were not easily accepted by people. The 1:3 group was located in the fourth quadrant, which meant that the excess extruded flour made the content of amylose in the batter, the degree of gelatinization, and the WAI increase, but the specific volume of the bread decreased, and the hardness, springiness, and chewiness properties decreased. The indicators for the 1:15, 1:12, and 1:9 groups of breads were closely grouped together and distributed around the origin, indicating that the results of the study were similar for the three groups of formulated rice flour, batter, and rice bread.

The results of the correlation plot are shown in Figure 8C. The red color on the graph represents a positive correlation and the blue color represents a negative correlation; the larger the value, the stronger the correlation [43]. The results reveal that the amylose content of the rice flour was significantly (*p* < 0.01) negatively correlated with the average area of gas cell and cohesion of the bread and was negatively correlated with the bread hardness and chewiness (*p* < 0.05). The gelatinization degree of the rice flour was significantly (*p* < 0.01) and positively correlated with the porosity, gas cell density, and organization of the bread, and positively correlated with the aroma of the bread. Saito et al. [44] pregelatinized rice flour with hot water and prepared rice bread, which increased the specific volume of the product by 27.09%. The degree of gelatinization of the rice flour was significantly (*p* < 0.01) negatively correlated with the average area of gas cell, hardness, cohesion, springiness, and chewiness of the bread. The specific volume of the bread was positively correlated (*p* < 0.01) with cohesion of the bread and appearance of the bread. The specific volume, porosity, color, and organization structure of the bread were positively correlated with the foaming stability of the batter. Fermentability positively correlated with the specific volume, porosity and appearance, taste, organization structure, and overall acceptability of the bread. The gas cell density of the bread negatively correlated with the average area of gas cell of the bread and textural properties such as hardness and chewiness. The aroma of the bread positively correlated with the foaming stability of the batter and the porosity and gas cell density of the bread, and it negatively correlated with the average area of gas cell and the hardness and textural properties of the bread, such as springiness and chewiness. The above results are relevant for predicting the baking quality of *japonica* rice bread.

## 3. Conclusions

This study analyzed the effects of extrusion treatment on the physicochemical properties, gelatinization, microstructure, and short-range molecular ordering of *japonica* rice flour. The results indicate that extrusion treatment increased the amylose content, gelatinization degree, and hydration capacity of the rice flour, while disrupting the starch granules and reducing the degree of molecular ordering and double helicity. The extruded flour was then mixed with *japonica* rice flour in varying ratios to prepare *japonica* rice bread. The addition of extruded flour improved the foaming properties, fermentability, and rheological characteristics of the batter, enhancing its viscoelasticity and ultimately influencing the baking quality of the bread. The 1:6 ratio group exhibited the best foaming stability, fermentability, specific volume, uniform pore structure, and reduction in hardness and chewiness. Additionally, the highest organoleptic scores for appearance, color, aroma, taste, texture, and overall acceptability were achieved in this group, making this bread the most preferred. Therefore, the best baking quality was obtained in this group.

The principal component analysis (PCA) revealed the relationships between the physicochemical properties of rice flour and batter and the baking quality of rice bread. The specific volume of *japonica* rice bread was positively correlated with the foaming properties of the rice flour and the leavening ability of the batter. Additionally, the properties of the rice flour and the fermentation capacity of the batter were positively correlated with the baking characteristics of the bread, while being negatively correlated with the average area of gas cell and the hardness, springiness, and chewiness properties. In conclusion, the extrusion treatment increased the amylose content, gelatinization degree, and hydration capacity of the *japonica* rice flour, thereby enhancing the fermentability of the *japonica* batter and improving the baking quality of the bread. These findings provide a basis for developing additive-free *japonica* rice bread.

## 4. Materials and Methods

### 4.1. Materials

“*Nan Japonica Rice 46*” (76.00% carbohydrate, 6.80% protein, 0.80% fat, and 16.40% moisture) was purchased from Fuhua Farm (Suzhou, China). Yeast was purchased from Angle Yeast Co., Ltd. (Yichang, Hubei, China). Salt, sugar, and butter were bought from the local market. The chemicals (hydrochloric acid, potassium hydroxide, iodine, potassium iodide) used were of analytical grade and were purchased from Sinopharm Chemical Reagent Co., Ltd. (Beijing, China).

### 4.2. Methods

#### 4.2.1. Preparation of Extruded Rice Flour

The preparation of extruded flour was derived using the methodology established in prior research [45]. *Japonica* rice was ground into flour and sieved through a 100-mesh sieve. The moisture content of the *japonica* rice flour was adjusted to 13% (*w*/*w*) with distilled water. The *japonica* rice flour was extruded with a TSX30/HN30-II twin-screw extrusion test machine (Jinan Runquan Machinery Equipment Co., Ltd., Jinan, Shandong, China). Three zones on the extruder were used to set the temperature. The barrel temperatures for zone 1, zone 2, and zone 3 were fixed at 60 °C, 100 °C, and 135 °C, respectively. The spindle frequency for each temperature was 35 Hz (screw speed, 227.5 rpm). Additionally, the feeding speed of the feed system was 15 Hz (screw speed, 97.5 rpm). After being air-dried at room temperature for 12 h, the extrudate was ground into flour and sieved through a 100-mesh sieve to be stored at 4 °C for later analysis.

#### 4.2.2. Preparation of Rice Flour

The rice bread was prepared from extracted rice flour and *japonica* rice flour in mass ratios (1:15, 1:12, 1:9, 1:6, and 1:3, *w*/*w*). And the rice bread prepared from *japonica* rice flour was used as a CK. Two hundred grams of rice flour, 40 g of sugar, and 3 g of salt were mixed, and then 4 g of yeast and 180 g of water were added to form a batter, followed by the addition of 10 g of butter and mixing. After that, the mixture was poured into molds and fermented for 1 h (35 °C, 85 °C humidity). Finally, the fermented batter was baked for 30 min (top at 150 °C, bottom at 180 °C). During the preparation process, rice batter or bread samples were taken for later analysis.

#### 4.2.3. Determination of Basic Physical and Chemical Indexes of Rice Flour

The total amylose and amylose contents of the rice flour were determined according to the kit instructions. Starch assay kit, product number 20240806; amylose assay kit, product number 20241023, purchased from Nanjing Jiancheng Bioengineering Institute (Nanjing, China).

#### 4.2.4. Determination of the Hydration Capacity of Rice Flour

The water absorption index (WAI) and water solubility (WS) of rice flour were determined as previously described [46]. Two grams of rice flour (*m*) was dispersed in 40 mL of distilled water, and the mixture was stirred in a water bath at 30° C for 30 min. Then, the mixture was centrifuged at 2148× *g* for 30 min and divided into the supernatant and wet precipitate (*m*_1_). The supernatant was dried at 105 °C (*m*_2_).

(1) $$WAI\left(\frac{g}{g}\right)=\frac{m_{1}}{m}\;$$

(2) $$WS/\%=\frac{m_{2}}{m}\times 100$$

where *m*_1_ denotes the mass of wet precipitate (g), *m*_2_ denotes the mass of dried solids of the supernatant, (g), and m denotes the mass of dried rice flour (g).

#### 4.2.5. Determination of the Degree of Gelatinization of Rice Flour

The degree of gelatinization of the rice flour was determined according the methods of Ge et al., with some modifications [47]. Fifty milligrams of rice flour was dispersed in 24.5 mL of distilled water, 0.5 mL of KOH solution (10 mol/L) was added, and the mixture was magnetically stirred for 5 min. Then, the mixture was centrifuged at 2148× *g* for 10 min. A total of 1.0 mL of the supernatant was added to 0.4 mL of HCl solution (0.5 mol/L) and mixed with distilled water to 10 mL. Finally, 0.1 mL of iodine solution (4 g potassium iodide dissolved in 100 mL distilled water, and then 1 g iodine dissolved in the above solution) was added. The absorbance of the reaction solution was measured at 600 nm (A_1_) with a UV-9600 spectrophotometer (Beijing Ruili Analytical Instrument Co., Ltd., Beijing, China). Another reaction system was prepared according to the above conditions, except the additions of distilled water, KOH solution, and HCl solution was 23.75 mL, 1.25 mL, and 1.0 mL, respectively. The absorbance was measured at 600 nm (A_2_). The degree of gelatinization was calculated as follows:

(3) $$Gelatinization\;Degree/\%=\frac{A_{1}}{A_{2}}\times 100$$

#### 4.2.6. Determination of the Microscopic Morphology of Rice Flour

Scanning electron microscopy (SEM) (QUANTA 250 FEG, Thermo Fisher Scientific, Waltham, MA, USA) was used to observe the particle morphology of the gold-sprayed rice flour at an accelerating voltage of 5.0 kV at magnifications of 1000× and 5000×.

#### 4.2.7. Determination of the Short-Range Ordered Structure of Rice Flour

The FTIR analysis was conducted according to a previous study with some modifications [25]. Rice flour was mixed with KBr (*w*/*w* = 1:100). The mixture was then milled and pressed into flakes. AN INVENIO-S FTIR spectrometer (Nicolet iS10, Thermo Fisher Scientific, MA, USA) was used to record the FTIR spectroscopy. The scanning wavenumbers ranged from 4000 cm^−1^ to 400 cm^−1^, and the mixture was scanned 64 times at a resolution of 4 cm^−1^.

#### 4.2.8. Determination of Rice Batter-Foaming Properties

Foaming properties were slightly modified using previously established methods [28]. Briefly, 5 g of batter freeze-dried flour and 28 mL distilled water (flour: water = 1:5.6, *w*/*w*) were mixed with a magnetic stirrer for at least 30 min at 25 °C in a glass beaker covered with Parafilm M. The mixture was then stirred for 2.5 min to form a foam (0 min), and the foam volume was measured at 0, 15, 30, 45, and 60 min.

#### 4.2.9. Determination of Fermentability of Rice Batter

Fermentability was slightly modified using previously established methods [47]. Briefly, 5 g of rice batter was placed into a 15 mL square tube with plastic wrap sealed at both ends and fermented for 60 min (35 °C, 85% humidity). The fermentation height of the rice batter was measured at 0, 15, 30, 45, and 60 min.

#### 4.2.10. Rice Batter Rheological Properties

The rheological properties of the rice batter (without yeast) were measured via a DHR dynamic rheometer (TA Instruments, Newcastle, DE, USA). The diameter of the parallel plate spindle was 25 mm. The gap size, strain, and frequency were 1 mm, 0.5%, and 1 Hz (within the viscoelastic region), respectively. The elastic modulus (*G′*) and viscous modulus (*G″*) of each rice batter sample were measured at 25 °C for 5 min, with frequency scanning ranging from 1~100 rad/s.

#### 4.2.11. Determination of the Specific Volume and Tissue Texture of Rice Bread

The volume of bread was determined according to the canola replacement method, and the specific volume was calculated from the ratio of the volume of bread (mL) to the weight of rice bread (g). The central area of the images of the rice bread slices (2 × 2 cm, 300 dpi) was selected and processed with the software JMJT I-image (Dongfu Jiuheng Instrument Technology Co., Beijing, China) for organization and organization structure analysis (porosity, gas cell density, and average gas cell area).

#### 4.2.12. Texture Profile Analysis of Rice Bread

The bread was sliced into 20 mm uniform slices and the whole organization structure pattern was determined using a TA.XTC-18 organization structure meter (Baosheng Industrial Development Co., Shanghai, China). The test parameters were as follows: probe was P/20; pre-test speed = 1.0 mm/s; test speed = 1.0 mm/s; post-test speed = 1.0 mm/s; degree of deformation = 50%; trigger force = 0.049 N; and compression interval = 5 s.

#### 4.2.13. Sensory Evaluation of Bread

Ten professionals trained in sensory evaluation were invited to conduct the sensory evaluation of bread. The acceptability of the bread was based on its appearance, color, aroma, taste, organization structure, and overall acceptability using a 9-point hedonic scale (9 = extremely like, 5 = neither like nor dislike, 1 = extremely dislike).

### 4.3. Statistical Analysis

All the experiments were carried out three times. The results are presented as the means ± SDs. SPSS 25.0 was used for data analysis, and one-way ANOVA (Duncan test) was used to analyze the differences between the mean values. Origin 2022 was used for graphic rendering, principal component analysis (PCA), and Pearson correlation analysis.

## Figures and Tables

**Table 1:** Effects of extrusion treatment on physicochemical properties and gelatinization degree of the rice flour.

Samples	Starch Content (%)	Amylose Content (%)	WAI (g/g)	WS (%)	Gelatinization Degree (%)
*Japonica* Rice Flour	77.50 ± 0.50 ^a^	8.69 ± 0.03 ^b^	2.66 ± 0.02 ^b^	2.29 ± 0.07 ^b^	6.67 ± 0.54 ^b^
Extruded Flour	74.38 ± 0.90 ^b^	9.77 ± 0.08 ^a^	5.71 ± 0.05 ^a^	19.55 ± 0.05 ^a^	88.26 ± 2.55 ^a^

^a, b^, Different letters in a column indicate significant (*p* < 0.05) differences. Values are expressed as mean ± SD (*n* = 3).

**Table 2:** Effect of extrusion treatment on the tissue texture of the rice breads.

Samples	Porosity (%)	Gas Cell Density (Number/cm^2^)	Average Gas Cell Area (mm^2^)
CK	33.80 ± 1.84 ^c^	4.50 ± 0.38 ^d^	83.60 ± 7.17 ^a^
1:15	42.71 ± 1.54 ^bc^	12.50 ± 1.42 ^c^	73.15 ± 2.39 ^a^
1:12	50.94 ± 3.30 ^ab^	14.25 ± 1.94 ^bc^	56.75 ± 4.70 ^bc^
1:9	47.36 ± 2.05 ^ab^	19.33 ± 2.05 ^ab^	68.88 ± 3.57 ^ab^
1:6	54.73 ± 1.80 ^a^	20.83 ± 0.55 ^a^	44.19 ± 3.25 ^c^
1:3	52.97 ± 4.51 ^a^	20.50 ± 2.74 ^a^	49.58 ± 2.70 ^c^

^a–d^, Different letters in a column indicate significant differences (*p* < 0.05). Values are expressed as mean ± SD (*n* = 3).

**Table 3:** Effect of extrusion treatment on the texture of the rice breads.

Samples	Hardness (N)	Cohesiveness	Springiness	Chewiness (N)	Resilience
CK	0.57 ± 0.02 ^a^	0.68 ± 0.01 ^b^	0.83 ± 0.01 ^a^	0.32 ± 0.01 ^a^	0.33 ± 0.01 ^ab^
1:15	0.38 ± 0.03 ^b^	0.68 ± 0.01 ^b^	0.79 ± 0.02 ^ab^	0.20 ± 0.02 ^b^	0.30 ± 0.01 ^ab^
1:12	0.36 ± 0.02 ^b^	0.72 ± 0.01 ^ab^	0.79 ± 0.03 ^ab^	0.22 ± 0.01 ^b^	0.30 ± 0.02 ^b^
1:9	0.35 ± 0.08 ^b^	0.73 ± 0.03 ^ab^	0.78 ± 0.04 ^ab^	0.20 ± 0.04 ^b^	0.33 ± 0.02 ^ab^
1:6	0.36 ± 0.07 ^b^	0.75 ± 0.01 ^a^	0.81 ± 0.02 ^ab^	0.22 ± 0.05 ^b^	0.35 ± 0.01 ^a^
1:3	0.26 ± 0.01 ^b^	0.14 ± 0.01 ^c^	0.73 ± 0.01 ^b^	0.14 ± 0.01 ^b^	0.29 ± 0.01 ^b^

^a–c^, Different letters in a column indicate significant differences (*p* < 0.05). Values are expressed as mean ± SD (*n* = 3).

## Data Availability

The original contributions presented in the study are included in the article; further inquiries can be directed to the corresponding authors.
